# Light exposure of roots in aeroponics enhances the accumulation of phytochemicals in aboveground parts of the medicinal plants *Artemisia annua* and *Hypericum perforatum*

**DOI:** 10.3389/fpls.2023.1079656

**Published:** 2023-01-19

**Authors:** Martina Paponov, Jörg Ziegler, Ivan A. Paponov

**Affiliations:** ^1^ Department of Horticulture, Norwegian Institute of Bioeconomy Research (NIBIO), Division of Food Production and Society, Ås, Norway; ^2^ Department of Molecular Signal Processing, Leibniz Institute of Plant Biochemistry, Halle, Germany; ^3^ Department of Food Science, Aarhus University, Aarhus, Denmark

**Keywords:** aeroponics, roots, LED – light emitting diode, Hypericum, Artemisia

## Abstract

Light acts as a trigger to enhance the accumulation of secondary compounds in the aboveground part of plants; however, whether a similar triggering effect occurs in roots is unclear. Using an aeroponic setup, we investigated the effect of long-term exposure of roots to LED lighting of different wavelengths on the growth and phytochemical composition of two high-value medicinal plants, *Artemisia annua* and *Hypericum perforatum*. In *A. annua*, root exposure to white, blue, and red light enhanced the accumulation of artemisinin in the shoots by 2.3-, 2.5-, and 1.9-fold, respectively. In *H. perforatum*, root exposure to white, blue, red, and green light enhanced the accumulation of coumaroylquinic acid in leaves by 89, 65, 84, and 74%, respectively. Root lighting also increased flavonol concentrations. In contrast to its effects in the shoots, root illumination did not change phytochemical composition in the roots or root exudates. Thus, root illumination induces a systemic response, resulting in modulation of the phytochemical composition in distal tissues remote from the light exposure site.

## 1 Introduction

The use of plant-based medicine is continuously growing (Thomson et al., 2014); however, our knowledge of the therapeutic efficacy of plant-based medicine products and their acute and chronic side effects in humans is limited. As a result, an increasingly urgent need has arisen for rigorous investigation of how plant-based medicine products affect human health (Ekor, 2014). The main challenge currently limiting this type of investigation is the availability of technology for growing medicinal plants with predictable concentrations of desired pharmaceuticals and phytochemicals. Indeed, most medicinal plants are cultivated in open fields, resulting in plants with varying concentrations of biologically active compounds due to effects from weather conditions and the soil environments (Borges et al., 2017). Obviously, clinical trials that use medicinal products of varying pharmaceutical and phytochemical compositions will not be reproducible or allow drawing of clear conclusions about the efficacy of plant-based pharmacological products.

This problem can be resolved by cultivating medicinal plants in protected agriculture (e.g., vertical farming), as this technology allows precise control of environmental conditions. Together with the selection of specific genotypes, this controlled plant growth will result in predictable accumulations of biologically active compounds in the biomass of cultivated medicinal plants for use in clinical trials. However, at present, the main focus in protected agriculture has been to optimize the environmental conditions that will maximize plant growth, with no focus on stimulating the accumulation of secondary metabolites. Unfortunately, the optimal conditions for plant growth necessarily exclude the imposition of any type of stress, but stress is necessary to stimulate the biosynthesis of secondary metabolites that have pharmaceutical benefits (Ramakrishna and Ravishankar, 2011). Conversely, extreme stress can have such a detrimental effect on plant growth that the resulting reduction in biomass also reduces the total yield of pharmaceuticals, despite the stimulating effect of stress on their concentrations. Thus, optimizing environmental conditions in protected agriculture should be directed toward enhancing the accumulation of secondary compounds without compromising plant biomass.

Indications that both enhanced accumulation of secondary compounds and efficient plant growth are possible simultaneously have come from experiments with Arabidopsis in which plant growth and the accumulation of secondary compounds were decoupled by the simultaneous activation of jasmonic acid (JA) signaling and deactivation of phytochrome B signaling pathways (Campos et al., 2016). Our previous investigation with *Hypericum perforatum* also supported the decoupling of growth and accumulation of secondary compounds by simultaneous activation of JA and deactivation of the phytochrome signaling pathways, respectively, as we saw a significant enhancement in the yield of pharmacologically active compounds by these treatments (Paponov et al., 2021a). Whether the application of other stress conditions or elicitors can facilitate this enhanced accumulation of secondary compounds without compromising plant growth is unknown and requires further investigation.

One stress that can induce the accumulation of secondary compounds is root illumination. The surprising observation that root exposure to light is a stress factor able to enhance the accumulation of secondary compounds, such as flavonols, was only recently discovered by comparing the traditional system of Arabidopsis cultivation in Petri dishes with similar dishes that allow the separation of environmental conditions for aboveground parts and roots by cultivating the roots in darkness (Silva-Navas et al., 2016; Lacek et al., 2021). The main motivation for those studies had been to evaluate the effects of illumination on root development and responses to different factors to understand why artificial Petri dish cultivation resulted in atypical plant responses not seen in natural conditions (i.e., when roots grow in the dark in soil). The conclusion of this investigation was that direct root illumination interferes with normal plant growth responses; therefore, the use of specific cultivation setups that provide darkness for the roots is important (Lacek et al., 2021; Miotto et al., 2021; Cabrera et al., 2022). Of relevance of the present proposal, these experiments identified illumination of roots as a new stress factor that can increase the accumulation of secondary compounds (Lacek et al., 2021); therefore, we hypothesize that exposure of roots to light can be used as a treatment in protected agriculture to stimulate the accumulation of secondary compounds in high-value medicinal plants.

In protected agriculture, plants are often cultivated substrate-free using hydroponics and aeroponics. Taking into account that water absorbs light, the mist systems used in aeroponics look especially promising for the cultivation of medicinal plants with root illumination because they will diminish the interference effect of water on light absorption by roots. A further advantage of aeroponic cultivation is that the roots, as well as substances exuded by the roots, can be easily harvested using a waste-free technology.

Studies with Arabidopsis have revealed several molecular mechanisms that explain the effect of root illumination on plant growth and development. First, light is perceived by photoreceptors, such as blue receptors, cryptochromes (CRY) and phototropins (PHOT), as well as by red/far-red receptor phytochromes (PHY) (Silva-Navas et al., 2015; Cabrera et al., 2022). These various photoreceptors are activated by specific wavelengths of light (Paik and Huq, 2019); therefore, exposing roots to different light wavelengths would be expected to promote the accumulation of different secondary compounds by the plants. Thus, investigations of the effects of specific light wavelengths on the accumulation of biologically active compounds are required.

Second, the effects of blue and white light (which contains the blue spectrum) on root growth and development are related to the generation of reactive oxygen species (ROS) (Yokawa et al., 2011). The induction of ROS by root illumination is also supported in protoplast experiments, where better yields of viable cells were obtained from dark-grown roots than from light-grown roots (Gonzalez-Garcia et al., 2020). ROS stimulate the biosynthesis of various secondary compounds that show anti-oxidative activity (Zandi and Schnug, 2022). Moreover, ROS interfere with intracellular trafficking (Zwiewka et al., 2015; Paponov et al., 2020), another important plant process that affects secondary metabolism (Roze et al., 2011) and therefore might also modulate the accumulation of desired pharmaceuticals and phytochemicals. ROS also play a key role in the development of root hairs, which are now viewed as impotant sites of secondary metabolite production (Dayan and Duke, 2003). However, the accumulation of secondary compounds, such as flavonols, can also decrease root hair formation (Gayomba and Muday, 2020). External application of another secondary compound artemisinin to Arabidopsis roots has also been shown to decrease both root hair density and root hair length (Yan et al., 2018). Therefore, negative feedback loops between the accumulation of secondary compounds and root hair formation can occur and could contribute to feedback regulation of the accumulation (stabilization) of secondary compounds when roots are exposed to light.

Third, root illumination responses can cross-talk with the signaling pathways of phytohormones, such as cytokinins and auxin. Specifically, root illumination stimulates the accumulation of flavonols, which are known to control auxin distribution at multiple levels, including the inhibition of auxin transport (Peer and Murphy, 2007) and increases in indole-3-acetic acid (IAA) catabolism (Peer et al., 2013). Flavonol-related reductions in auxin levels can stimulate the biosynthesis of cytokinins (Nordstrom et al., 2004) that in turn, stimulate flavonol biosynthesis (Silva-Navas et al., 2016). Thus, a complex auxin–cytokinin–flavonol network might be involved in the regulation of root growth, the formation of root hairs, and the accumulation of secondary compounds.

Fourth, in addition to the local effects of root illumination, as observed in numerous investigations, root illumination also has a systemic effect, as it can enhance shoot growth and the accumulation of anthocyanins in the shoot (Silva-Navas et al., 2015).

Among medicinal plants, the genera *Artemisia* and *Hypericum* are considered especially important producers of highly valuable compounds. *Artemisia annua* produces the sesquiterpene lactone artemisinin, which is currently believed to be the most effective anti-malarial drug available (Chadwick et al., 2013). *Hypericum perforatum* produces antidepressant naphthodianthrones (hypericin and pseudohypericin), flavonoids, and other phenolic compounds (Mir et al., 2019). The biosynthesis of these compounds in *Artemisia* and *Hypericum* has awakened an interest in cultivation of these plants in protected agriculture using hydroponics (Murch et al., 2002; Koehorst et al., 2010). However, further investigations are needed to enhance the accumulation of biologically active compounds in these plants cultivated in protected agriculture.

Cultivation of medicinal plants hydroponically or aeroponically allows the harvesting of highly valuable medicinal compounds from the plant biomass as well as from root exudates. The collection of biologically active compounds from exudates has a distinct advantage, as the exudates can be collected non-destructively over the lifetime of the cultivated plants, and these compounds can be separated far more easily from the hydroponic solution than from the intact plant tissue. Because elicitors can enhance the biosynthesis and exudation of valuable compounds, the effect of root illumination should be evaluated for both the accumulation of these compounds in the above- and below-ground parts, as well as in root exudates.

The aim of this work was to estimate the effect of root illumination at different light wavelengths on plant growth, accumulation of biologically active compounds in plant biomass, and their exudation by roots in two medicinal plants, *A. annua* and *H. perforatum*. We also estimated the effect of root illumination on root hair development, assuming that modulation of the accumulation and/or exudation of biologically active compounds might be related to root hair formation.

## 2 Materials and methods

### 2.1 *Artemisia annua* plant material and experimental performance

**Figure 1:**
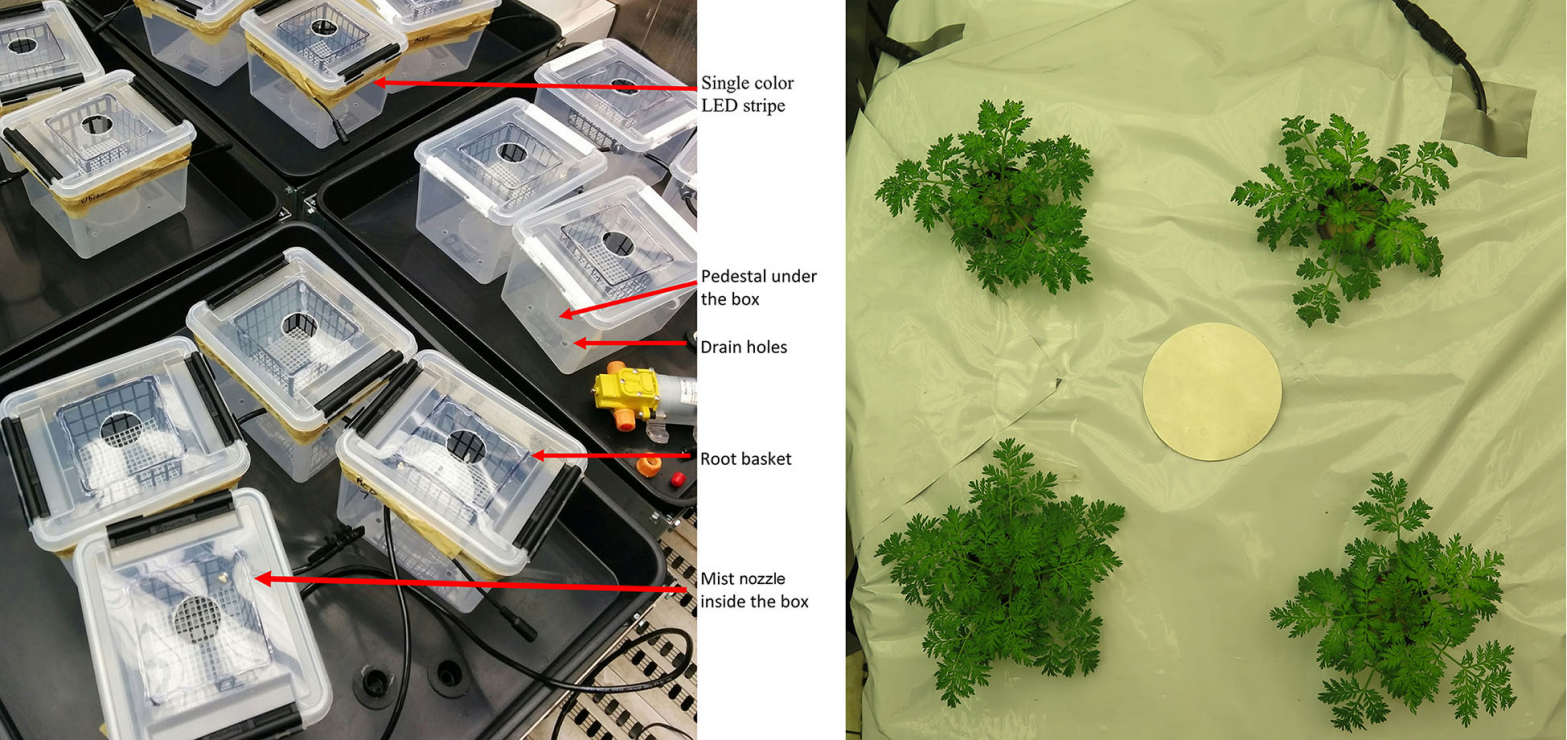
The experimental setup. The roots of *Artemisia annua* and *Hypericum perforatum* were illuminated continuously under different unicolored wavelength by flexible light-emitting diode (LED)-stripes fixed outside a transparent plastic container in a high pressure aeroponics system for 14 and 19 days, respectively **(A)**. The 4 replications were covered with light-impermeable foil including the tray that captured the drained nutrient solution **(B)** to investigate the effect of the root illumination stimulus on plant developmental parameters, root hair density and length and the enhancement of secondary metabolite accumulation.

Seeds of *Artemisia annua* L., purchased from Anamed International (https://anamed.org, Winnenden, Germany), were sterilized by incubation in 70% ethanol for 10 min. The sterilized seeds were sown in “sandwich” filter paper placed between mat layers (Clas Ohlson, Insjön, Sweden) and fixed in plastic plates at 1 mm intervals in a line just 1–2 mm below the top of the filter paper. The filter paper was soaked with 50% Hoagland nutrient solution (NS) (Kaya et al., 2000) containing 500 µM KNO_3_. The *A. annua* plants were also provided with a fresh and continuous flow of 50% Hoagland NS to wash out the autotoxic exudations of the *A. annua* seedlings. The seedlings were protected from light until the first leaves emerged. At 15 days after sowing (DAS), the largest *A. annua* seedlings had a 1 cm long root, and these *A. annua* plants were transplanted in a 5 mm thin sliced sponge and placed into the hydroponics system containing 50% Hoagland NS at 4 plants per 2 L pot. At 26 DAS, the *A. annua* plants were transferred to pots (one plant per pot) containing 650 mL of continuously aerated 100% Hoagland NS. The pots were covered with an impermeable plastic cover, and the plants were acclimatized until 31 DAS. The plants were then transplanted to a self-built high-pressure aeroponics system that allowed provision of different light spectra to the roots (**Figure 1**). Four plants used as controls were retained in the “water culture” hydroponic treatment. The root light treatments were initiated at 31 DAS. The *A. annua* roots were treated with light for at least 14 days, and plants were harvested at 45, 46, 47, and 48 DAS.

### 2.2 *Hypericum perforatum* plant material and experimental performance

Seeds of St. John’s wort (*Hypericum perforatum* L.; Hypericaceae), purchased from Rarexoticseeds (https://www.rarexoticseeds.com/, accessed on 28 September 2022), were sterilized in 2.5% sodium hypochlorite for 10 min and then washed thoroughly 5 times with deionized water. The sterilized *H. perforatum* seeds were germinated in “sandwich” filters, as described for Artemesia. Seeds were sown at 5 mm intervals in a line 2–3 mm below the top of the filter paper and incubated in a 10% NS containing 500 µM KNO3. The full NS contained 1 mM CaSO_4_, 1 mM K_2_HPO_4_, 1 mM KH_2_PO_4_, 2 mM MgSO_4_ (Carlisle et al., 2012), and micronutrients with the following concentrations: 15 µM Fe, 10 µM Mn, 5 µM Zn, 30 µM B, 0.75 µM Cu, and 0.5 µM Mo. For the first 2 days, the seeds were kept in darkness at 18°C. Seedlings were then transferred at 14 DAS from the “sandwich” system to a water culture with 7 plants in a 2 L pot containing 50% NS and 2.5 mM KNO_3_ and fixed with foam slabs onto the pot lid. At 26 DAS, the plants were transplanted into a hydroponic sponge and fixed onto the lid of the self-built high-pressure aeroponic system (**Figure 1**). After this transplantation, the plants were cultivated in 100% NS with 2.5 mM KNO_3_. After 4 days of adaptation (i.e., at 30 DAS), the roots were exposed to light treatments as described for *A. annua* above, resulting in 4 different light spectrum treatments and two control treatments in darkness at different root temperatures.

### 2.3 Root light treatments for *A. annua* and *H. perforatum* plants

The setup for the cultivation of *A. annua* and *H. perforatum* is described in **Figure 1**. About 1 cm below the edge of the plastic box, a single colored, cuttable LED strip (60 LED/m; working voltage: 12 V) about 72 cm in length was attached to the outside. In the experiment with *A. annua*, we studied the effects of root exposure to white (400–700 nm), blue (465 nm), and red (630 nm) light on plant growth, dry matter allocation, formation of root hairs, and accumulation of artemisinin in the roots and shoots of the aeroponically grown plants. The measured light intensities close to the roots were 16.6 ± 0.4, 1.5 ± 0.1, 19.3 ± 0.7 µmol m^-2^ s^-1^ photosynthetically active radiation (PAR) for white, blue, and red wavelengths, respectively. In the experiment with *H. perforatum*, we studied the effects of root exposure to the same light conditions, but we also included a green light (532 nm) treatment, as well as an increased nutrient solution (NS) temperature treatment, on plant growth, accumulation of secondary compounds in biomass, and exudation of secondary compounds by the roots. The light intensity close to the roots was 10.1 ± 0.2 µmol m^-2^ s^-1^ PAR for the green light treatment. The increased temperature treatment was an additional control used to distinguish whether the effects of lights on roots were due to emitted photons or to enhanced temperature. All treatments were replicated four times.

### 2.4 Growth chamber conditions for *A. annua* and *H. perforatum*

During the entire cultivation period, the conditions in the growth chamber were maintained at a 16 h/8 h day/night photoperiod (8:00–24:00 light) at a light intensity of PAR 190 µmol m^-2^ s^-1^, 22°C/18°C day/night temperature, 60/80% day/night air humidity, and atmospheric CO_2_ concentrations.

The pH of the NS was monitored regularly, and controlled between pH 5.5 and 6.3 for *A. annua* and 6.3–6.9 for *H. perforatum*. The temperature of the root environment was controlled daily and ranged between 31.4 and 32.7°C for light treatments and at about 25.1°C for the dark control. In the case of *H. perforatum*, an additional control with an elevated temperature was included to achieve a comparable root environment temperature by warming the NS to 35.5°C with a fully submersible aquarium heater thermostat (KD Heater Co., Ltd.) prior to the mist supply. Under this condition, the temperature in the rhizosphere of the dark control plants was close to 32°C.

### 2.5 Final sampling of *A. annua* and *H. perforatum*

The total fresh weights of the shoot and root of *A. annua* were recorded at 45–47 DAS. The total fresh weight of the leaves, stem, and roots of *H. perforatum* were recorded at 49 DAS. An aliquot of about 0.9 g of fresh shoot or root material was weighed and immediately frozen in liquid nitrogen in a pre-weighed 5 mL Eppendorf tube and vacuum lyophilized for 36 h (leaves) or 24 h (roots) in a BK-FD10S freeze-dryer (BIOBASE, Jinan, China). After determining the dry matter % of the samples, the shoot and root materials were powdered (Star-Beater VWR with 5 mm metal balls, 29 Hz for 3 min) to a fine dust and stored at −80°C until further processing.

The total dry biomass, ratio of root weight to total plant weight (RWR), and the shoot and root dry matter percentages (SDM% and RDM%) were determined for *A. annua*. For *H. perforatum*, recording the LDM% was possible, as the stem could be taken apart. A further 0.3–0.5 g of root material was collected, weighed, and preserved in 50% ethanol for further analysis of the root hairs.

### 2.6 Analysis of roots and root hairs of *A. annua* and *H. perforatum*

The root samples were mounted in water and visualized with an Olympus CX-41 microscope (Olympus Corporation, Tokyo, Japan) and dark-field illumination. Images were captured with an ocular-mounted Toupcam U3CMOS 5.1 MP camera (ToupTek Europe, Stansfield, United Kingdom) using ToupView 3.7 software. The average density (hairs/mm) and length (mm) of the root hairs were determined using Fiji software. The dataset is based on the measurements of 20 root segments per plant, of which 10 segments were from the root tip and 10 segments were from the corresponding differentiated root area. This resulted in a total of 142–163 root hair length measurements for the four replicates per treatment for *A. annua*.

For *H. perforatum*, 10 randomly chosen recordings of root segments per plant were investigated for root hair density and root hair length measurement, resulting in 39–46 segments per treatment and about 12 root hair length measurements per segment.

### 2.7 Extraction of bioactive compounds from leaves and roots of *A. annua* and *H. perforatum*

A 100 mg shoot and 25–75 mg of a lyophilized and powdered root samples (prepared by treatment at 29 Hz for 3 min with a Starbeater device [VWR, Radnor, PA, USA]) of *A. annua* or *H. perforatum* were vortexed for 20 min at maximal speed in 2 mL Eppendorf tubes containing a steel bead (5 mm diameter) and 1.5 mL 80% methanol. The extract was centrifuged for 5 min at 17,000 × *g* and the supernatant was collected. The supernatant was centrifuged again to prevent later sedimentation. The clean supernatant was stored at −20°C until it was analyzed by ultra-high-performance liquid chromatography (UHPLC).

### 2.8 Exudate collection from *H. perforatum* roots

The root exudates were collected from plants by collecting the drained NS for 10–12.5 h at night for four sequential days (46, 47, 48 DAS for 4 plants and 49 DAS for 3 plants). For exudate collection in distilled water, each plant for all treatments was transported to a pot covered by light-impermeable foil and containing 550 mL continuously aerated distilled water (pH 6.2) 11–12.5 h during the day. After collection of exudates in distilled water, the plants were sampled.

The NS or distilled water containing root exudates was prefiltered using a Sigma-Aldrich^®^ vacuum filtration assembly (Z290432-1EA, Merck, Darmstadt, Germany) and Nalgene bottle-top sterile filters (45 mm diameter and 0.45 μm pore size) (Z370533, Merck, Darmstadt, Germany). The amount of drained NS was recorded before and after exudate extraction. Approximately 500–1000 mL of the filtered NS and 500 mL of distilled water-exudate solution were loaded onto Bond Elut™ C18 (Agilent Technologies, Santa Clara, CA, USA) solid-phase extraction cartridges with a 1 g bed mass and 40 µm particle size to trap the non-polar and semi-polar secondary compounds. Columns were activated with 2 mL 100% MeOH (10516279, Fisher Scientific, Waltham, MA, USA), followed by 2 mL 1% aqueous formic acid (33015, Fluka, Honeywell, Morris Plains, NJ, USA). The columns were washed with 2 mL distilled water, and the hydrophobic compounds were eluted with 2 mL of 2% formic acid in MeOH. The eluent was stored at −20°C until analysis of total phenolics and the UHPLC analyses for the leaves, roots, and root exudates.

### 2.9 Assay of total phenolic content

Total phenolic content was estimated in root exudates of *H. perforatum* using the Folin–Ciocalteu assay (Ainsworth and Gillespie, 2007). The exudate extract derived from NS was diluted 1:2 (v/v) before analysis. In brief, 200 µL of the diluted NS root exudate solutions or 200 µL of the distilled water root exudate solutions were combined with 200 µL of a 10% Folin–Ciocalteu (F–C) reagent (F9252, Merck, Darmstadt, Germany). An 800 µL volume of 700 mM Na_2_CO_3_ (S7795, Merck, Darmstadt, Germany) was added, and the samples were incubated at room temperature for 2 h in darkness. Triplicate samples were then transferred to a spectrophotometry plate reader (Multiscan GO, Thermo Fisher Scientific, Waltham, MA, USA), and the absorbance was measured for each well at 765 nm at room temperature. Measurements were standardized against gallic acid (48630, Merck, Darmstadt, Germany) (50 μM–2.5 mM in 80% MeOH). The root exudation rate of total phenolics into the 500–1000 mL water or NS was calculated based on the total gallic acid equivalents measured in the 100 or 200 µL concentrated extract fraction. The rate of exudation was expressed as the amount of total phenolics per FW of roots and the duration of exudation.

### 2.10 Quantitative determination of artemisinin

UHPLC-MS/MS analysis was performed using an Agilent 1290 LC system (Agilent, Waldbronn, Germany) connected to an API 3200 triple quadrupole mass spectrometer by a TurboIon source (AB Sciex, Darmstadt, Germany). Artemisinin was separated on a Nucleoshell C18 column (2.6 µm, 50 x 3 mm; Macherey-Nagel, Düren, Germany) at 30°C at a flow rate of 500 µl min-1 using 0.02% (v/v) acetic acid in water or in acetonitrile as eluents A and B, respectively. The percentage of B was linearly increased from 15% to 98% within 7 min, held at 98% B for 2.5 min, and then returned to the starting conditions within 0.5 min. The column was then re-equilibrated for 2 min. The ion source was operated in the positive mode at a curtain gas pressure of 30 psi, an ion source voltage of 5,500 V, a temperature of 450°C, and a sheath and de-solvation gas pressure of 50 psi. Data were acquired in the multiple reaction monitoring mode (Q1 and Q3 set at unit resolution) with target scan time of 50 ms. Quantifier and qualifier transitions for each compound as well as compound specific instrument parameters are shown in **Supplementary Table S1**. The IntelliQuant algorithm of the Analyst 1.6.2 software (AB Sciex, Darmstadt, Germany) was used to integrate the peaks for artemisinin. Metabolite concentrations were calculated using an artemisinin standard curve in the range of 0 to 5.6 µg ml^-1^ and divided by the fresh weights. Dilution and injection volumes of the samples were adjusted according to the linear range of the artemisinin standard curve.

### 2.11 Quantitative determination of secondary compounds by UHPLC

The secondary compounds were analyzed by UHPLC (1290 Infinity II, Agilent Technologies, Santa Clara, CA, USA) with a diode array detector and an electrospray ionization single-quadrupole detector (6120 SQ, Agilent Technologies, Santa Clara, CA, USA). Separation was achieved on an Ascentis Express C18-column (100 × 2.1 mm, 2 µm, Supelco, Merck, Darmstadt, Germany). A gradient with increasing amounts of acetonitrile (solvent B) in 0.02% formic acid (solvent A) was used as follows: from 2% to 5% B (in 1 min), from 5% to 33% B (in 6 min), from 33% to 95% B (in 10 min), from 95% to 100% B (in 3 min), and finally from 100% to 2% B (in 1 min). Column reconditioning was achieved using a post-time of 2 min. The flow rate was set to 0.3 mL/min, and injections were 10 µL. All samples were filtered (0.45 µm) prior to analysis. Mass spectra were acquired in scan mode (180–700, *m*/*z*) with a scan time of 500 ms, fragmentor voltage at 50 V, and both positive and negative modes of ionization. The source was operated with a gas temperature at 300°C, gas flow at 7.0 L/min, nebulizer pressure 30 psi, and capillary voltage at ±3 kV.

The secondary compounds were characterized based on co-chromatography with authentic samples and by their UV-Vis absorbance spectra, as well as by their pseudo-molecular and fragment ions, according to previous reports (Tatsis et al., 2008; Tusevski et al., 2013; Porzel et al., 2014).

Quantifications were made based on the UV-Vis absorbance at the detection windows of 280, 360, and 590 nm for catechins, flavonols, and naphtodianthrones (NDAs), respectively. Standard curves were prepared for each group of phenolic compounds using flavan-3-ol (–)-epicatechin (Merck, Darmstadt, Germany) for catechin, epicatechin, and procyanidin dimer; chlorogenic acid (phenolic acid) (Merck, Darmstadt, Germany) for chlorogenic acid and coumaroylquinic acid; rutin and isoquercitrin (PlantChem, Eiken, Norway) for flavonols; and NDA pseudohypericin and hypericin (Merck, Darmstadt, Germany) for those compounds.

### 2.12 Statistics

Data were statistically analyzed by analysis of variance (one-way ANOVA). The treatments were replicated four times. When significant treatment effects were indicated by ANOVA, Fisher’s protected LSD test was used to compare the individual means (Statistica 13 software package, Palo Alto, CA, USA).

## 3 Results

### 3.1 Effect of root lighting on plant growth traits and artemisinin accumulation in shoots and roots of *Artemisia annua*

Light exposure of the root was considered an elicitor of a stress response in the roots that could induce both plant growth reduction and enhanced accumulation of biologically active compounds. Both plant growth (biomass accumulation) and enhanced concentration affect the total yield of biologically active compounds. Therefore, we estimated the effect of root illumination on plant growth traits and concentration of artemisinin in the shoot and roots. As an additional control, we included a treatment with water culture.

**Figure 2:**
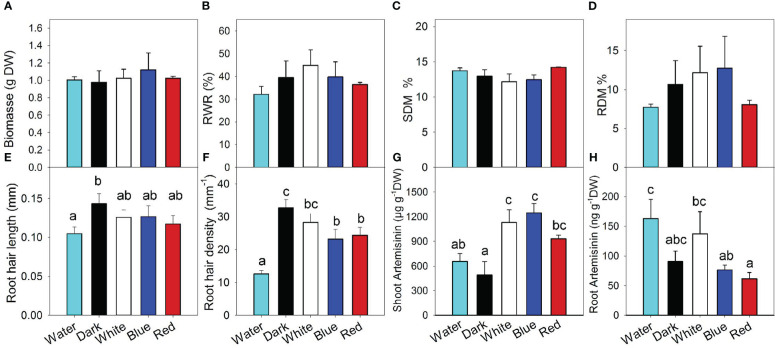
The effect of the exposure of *Artemisia annua* roots to white, blue, and red light on plant biomass **(A)**, root weight ratio (RWR) **(B)**, shoot dry matter content (SDM) **(C)**, root dry matter content (RDM) **(D)**, root hair length **(E)**, root hair density **(F)**, and accumulation of artemisinin in the shoot **(G)** and roots **(H)**. “Water” treatment corresponds to water culture hydroponics with continuous aeration. Plants in all other treatments were cultivated in aeroponics. Differences between means with different letters are statistically significant at p<0.05 (*n=4*). Roots of *A. annua* were exposed to the light at 31 days after sowing (DAS). The final plant samples were collected at 48 DAS.

**Figure 2** shows that different root light treatments did not affect plant growth (**Figure 2A** and **Figure S1**), nor did they affect dry matter allocation to the roots (**Figure 2B**) or the dry matter content in the shoot and roots (**Figures 2C, D**). This indicated that root illumination had no negative effects on plant growth, even though root illumination is considered a stress factor (Yokawa et al., 2014).

As expected, high-pressure aeroponics with the roots in the darkness increased root hair length (**Figure 2E** and **Figure S1**) compared with water culture. Lighting with white, blue, and red wavelengths tended to decrease root hair length; however, this effect was not statistically significant. High-pressure aeroponics had an even stronger effect on root hair density (**Figure 2F**) than on root hair length, increasing the root hair density almost threefold. Blue and red light reduced the root hair density by 30%. When blue and red lights were combined through the application of white light, no additive effects on the inhibition of root hair density were found. The absence of an additive effect indicates that the same signal pathway was activated by both blue and red light.

The different types of hydroponic systems did not significantly affect the concentration of artemisinin in the shoot; however, the exposure of the root to white and blue light enhanced artemisinin accumulation more than twofold. The effect of red light on artemisinin accumulation in the roots tended to be weaker than white and blue light, indicating that blue light might have a supplemental stimulating effect on artemisinin accumulation in the shoot; however, the difference was not statistically significant (**Figure 2G**).

As expected, roots accumulated several thousandfold less artemisinin than shoots (**Figure 2H**). Surprisingly, the cultivation of *A. annua* in an aeroponics system compared to water culture tended to decrease artemisinin concentration in the roots, despite the increases in root hair length and density in the aeroponic system. Red and blue light did not change the accumulation of artemisinin in the roots compared to the aeroponics control; however, white light tended to increase the artemisinin content.

### 3.2 Effect of root lighting on accumulation and exudation of secondary compounds in *Hypericum perforatum*

**Figure 3:**
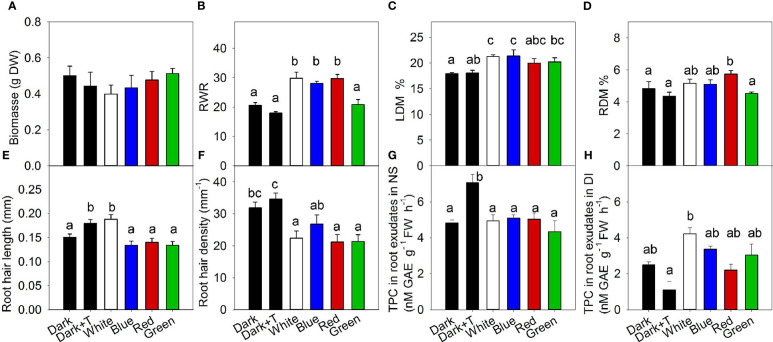
The effects of the exposure of *Hypericum perforatum* roots to white, blue, red, and green light and to a higher nutrient solution temperature (35.5°C) on plant biomass **(A)**, root weight ratio (RWR) **(B)**, leaf dry matter content (LDM) **(C)**, root dry matter content (RDM) **(D)**, root hair length **(E)**, root hair density **(F)**, and amount of total phenolic compounds (TPC) in root exudates during plant cultivation in nutrient solution **(G)** and amount of TPC released into distilled water shortly before the final sampling **(H)**. Differences between means with different letters are statistically significant at p<0.05 (*n=4*). Roots of *H. perforatum* were exposed to the light at 30 days after sowing (DAS). The final plant samples were collected at 47 DAS.

In agreement with the results obtained with *A. annua*, root exposure to different light wavelengths did not modulate plant growth of *H. perforatum* (**Figure 3A**), indicating that the efficacy of this stress factor was not sufficient to inhibit plant growth. Despite the absence of differences in growth, root exposure to white, blue, or red light enhanced dry matter allocation to the roots (**Figure 3B**). The fact that green light did not enhance dry matter allocation to the roots indicates the dependence of this response on blue and red receptors (**Figure 3B**). Enhanced accumulation of the percentage of leaf dry matter in *H. perforatum* following exposure of the roots to white and blue light (**Figure 3C**) indicated a less efficient utilization of non-structural carbohydrates for growth, indicating that root lighting is only a weak stress in plants. Red light also increased dry matter content in roots (**Figure 3D**), supporting a role for the phytochrome photoreceptor in this process.

White light increased root hair length; however, this increase seemed to be due to the higher temperature due to exposure of the roots to LED because a similar effect on root hair length was observed in the control treatment with increased temperature without lighting (darkness) (**Figure 3E**). The facts that all LED lighting treatments, independent of specific wavelengths, increased the temperature in the roots and that a specific single wavelength decreased root hair length compared with white light or the dark control with enhanced temperature led us to suspect a complex interference of a specific light wavelength with temperature. Root hair density was not changed by temperature; however, it was strongly reduced by white light (**Figure 3F**). A similar effect was observed for the inhibition of root hair density induced by red and green light; however, the blue light effect was weaker.

Higher temperature increased phenolic exudation, as indicated by the estimation of total phenolics in root exudates during root cultivation in nutrient solution. However, all light treatments did not change the level of total phenolics in root exudate (**Figure 3G**). Transferring the plants to distilled water before the final plant biomass sampling showed an opposite effect of cultivation temperature on phenolic exudation, thereby the higher temperature treatment reduced phenolic exudation in distilled water. This reduced exudation in distilled water might indicate a compensation for the higher exudation during cultivation in nutrient solution. All light conditions increased or tended to increase phenolic exudation into distilled water, with the strongest effect observed for the white light (**Figure 3H**)

**Figure 4:**
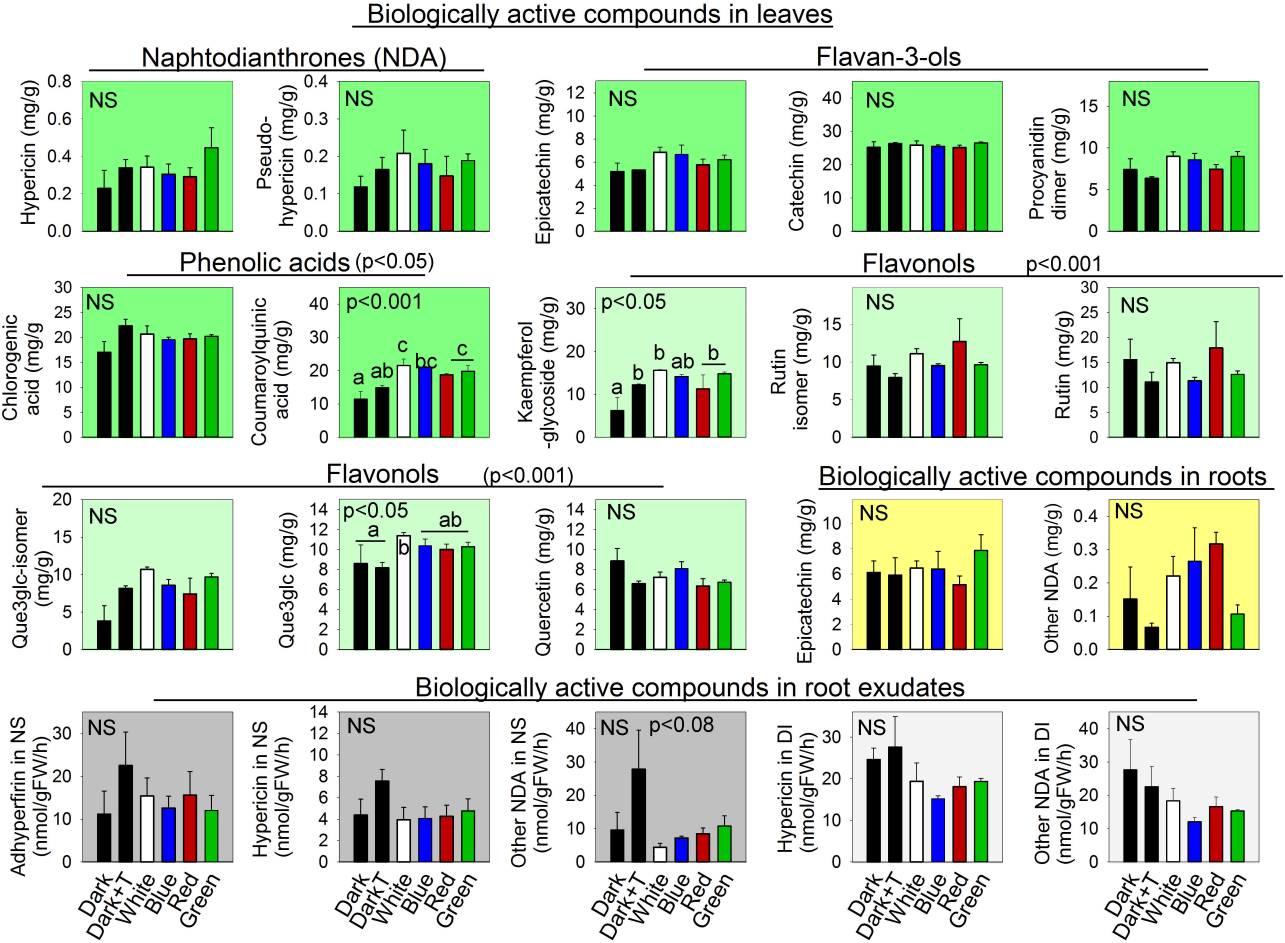
The influence of different light spectra and increased temperature of the nutrient solution on the concentration of biologically active compounds in leaves and roots and on the rates of root exudation of those compounds in *Hypericum perforatum* cultivated in an aeroponics system. Values are means ± SE. NS is not significant at the 0.05 level probability test (one-way ANOVA) (*n=4*). The data were analyzed using the LSD test. Differences between means with different letters are statistically significant at the 0.05 probability level. Abbrev.: naphtodianthrone (NDA).

Among the measured secondary compounds, root exposure to any light spectrum enhanced the accumulation of coumaroylquininic acids in the aboveground parts of *H. perforatum*. By contrast, increased temperature alone did not induce a similar effect, indicating that this effect on the accumulation of secondary compounds in shoots is light specific (**Figure 4**).

White light increased the accumulation of total flavonols in the shoots, although an increase in temperature alone did not. Root exposure to red, blue, and green light also enhanced the accumulation of flavonols; however, these effects tended to be weaker than the effect of white light, indicating a potential additional effect of different wavelengths on the accumulation of flavonols in the shoots. Among specific flavonols, root illumination significantly increased accumulation of kaempferol-glycoside and quercetin 3-galactoside. No significant differences were noted in the accumulation of secondary compounds in roots or in secondary compound exudation between the dark control and light-treated roots.

## 4 Discussion

In natural environments, roots grow in darkness (Tester and Morris, 2006). Consequently, the exposure of roots to light induces a stress response that induces changes in root growth and development, plant hormone balances, and secondary metabolism, which together help roots avoid direct illumination. The main finding of our work is that root illumination can enhance the accumulation of secondary compounds in the aboveground parts of high-value medicinal plants. Thus, root illumination induces a systemic response, as the modulation of the phytochemical composition occurs in distal tissue remote from the light exposure site. This finding was consistently observed in both medicinal species, as root exposure to white, blue, or red light enhanced the accumulation of artemisinin in the shoot of *A. annua* (**Figure 2G**) by 2.3, 2.5, and 1.9 fold, respectively, and white, blue, red, and green root illumination increased the leaf concentrations of coumaroylquinic acid in *H. perforatum* by 89, 65, 84, and 74%, respectively (**Figure 4**). Root lighting also increased the accumulation of flavonols in *H. perforatum* leaves. In contrast to its effect in shoots, root illumination had a weaker effect on the phytochemical composition in the roots or root exudates, and no statistically significant differences were identified.

Previous studies have extensively reported the effects of root illumination on the physiological and molecular responses of roots (Yokawa et al., 2014; Silva-Navas et al., 2015; Kumari et al., 2019; Miotto et al., 2021), whereas the effects of root illumination on the growth and metabolism of the aboveground plant parts have received less attention. Most previous investigations were also carried out on Arabidopsis (Yokawa et al., 2014; Silva-Navas et al., 2015; Kumari et al., 2019; Miotto et al., 2021). In plant roots, direct effects of root exposure to light were identified for primary root growth, secondary root development, root hair formation, accumulation of flavonols, and root tropic responses (Lacek et al., 2021). In plant shoots, root illumination was reported to increase shoot growth and enhance the accumulation of anthocyanin (Silva-Navas et al., 2015), indicating the presence of a systemic effect of root illumination on growth and secondary metabolism in Arabidopsis. However, the mechanism of action of this systemic effect and whether it occurs in other plant species has not been further investigated.

Two different hypotheses could explain the systemic modulation of the phytochemical composition in the shoot in response to root illumination. The first hypothesis is that a root-derived signal induced by root light exposure is transported to the shoot, where it activates secondary metabolism. The second hypothesis is that the biosynthesis of secondary compounds is induced in the roots by root illumination, and the compounds are then transported to the shoot.

### 4.1 A root-derived signal stimulates the accumulation of secondary compounds in the shoot

This hypothesis appears to be the most promising of the two. However, we were unable to identify which photoreceptors might be involved in this response because this response was not restricted to any specific light wavelength; instead, all the tested wavelengths induced it. The absence of differences between different wavelengths on the accumulation of phytochemicals in plants and the observation that every wavelength of LED lighting increased the temperature in the aeroponic pots (in which the plant roots were localized), led us to assume that the observed response might not be related to illumination but was instead a response to increased temperature in the pots, independent of the wavelength. We test this assumption by including an additional control in the *H. perforatum* experiment, in which the temperature of the nutrient solution was warmed to the temperature of the solution in the pots measured in the light treatments. Our finding that warming alone did not increase the accumulation of several biologically active compounds in the shoot confirmed that the root response was independent of temperature and was truly related to light perception by the roots. The similarity of effects of different wavelengths and the absence of additive effects between red and blue light when supplied in combination, as white light, indicated a common mechanism of light action on the accumulation of phytochemicals in the aboveground part of plants.

Different root phenotypes are induced by blue or white light versus darkness, red, or green light in Arabidopsis and could reflect the capacity of blue light and white lights (which contains the blue spectrum) to enhance reactive oxygen species (ROS) production in the roots (Yokawa et al., 2011). ROS are directly involved in systemic signaling, as ROS waves can move at rates exceeding 8.4 cm per minute (Miller et al., 2009); therefore, ROS could be a potential root-derived signal that could move from the root to the shoot. However, the lack of any significant difference between white and red light on the accumulation of artemisinin in the *A. annua* shoot or on the accumulation of secondary compounds in the *H. perforatum* shoot leads us to assume the systemic phytochemical accumulation observed in the shoots might occur independent of the light stimulating production of ROS in the roots in our experiments.

The presence of a green light–specific response in our experiment with *H. perforatum* is difficult to interpret, given that current evidence indicates that green light responses in plants rely on the residual perception of these wavelengths primarily by red and blue photoreceptors (Battle et al., 2020). However, this green light response also appears unrelated to ROS production, as Arabidopsis roots show different phenotypic responses in growth and development in response to root exposure to blue or green lighting (Silva-Navas et al., 2015). Thus, the response observed in the present study following illumination of roots with green light also supports that the systemic accumulation of biologically active compounds in *H. perforatum* shoots might occur independent of the light stimulating production of ROS in the roots.

Other types of root-derived signals that can be transmitted to the shoot are plant hormones, as these are known to play a key role in shoot–root communication. The major hormones produced in the roots and then transported to the shoots include growth-stimulating cytokinins (Kiba et al., 2013) and growth-inhibiting hormones, such as abscisic acid (ABA), jasmonic acid (JA), salicylic acid (SA), and the ethylene precursor 1-aminocyclopropane-1-carboxylic acid (ACC) (Paponov et al., 2021b). A previous investigation of Arabidopsis showed that root illumination could enhance the shoot growth rate, indicating a potential role for cytokinins. In our study, root illumination did not change shoot growth, suggesting that hormonal changes were not sufficiently strong to alter plant growth and dry matter allocation between the roots and the shoot. In Arabidopsis, experiments have shown the importance of cytokinin signaling in root phototropism (Silva-Navas et al., 2016), and enhanced cytokinin transport from root to shoot is able to stimulate flowering, suggesting that root illumination might increase cytokinin transport from the root to shoot to induce early flowering (D'aloia et al., 2011; Silva-Navas et al., 2015).

In *in vitro* culture experiments, cytokinins are widely used as stimulators to enhance the production of phytochemicals (Honig et al., 2018), further supporting a potential role for cytokinins in the root illumination response. However, we cannot exclude the possibility that other hormones, such as ABA, JA, SA, and ethylene, that are also known to stimulate secondary plant metabolism may also be involved in systemic signaling due to root illumination and the increased accumulation of phytochemicals in the shoots.

### 4.2 Root illumination does not modulate the accumulation of biologically active compounds in the roots and root exudates

The second hypothesis was that root illumination enhanced the biosynthesis of secondary compounds in the roots and subsequent transport and accumulation in the aboveground parts. If this hypothesis were correct, we would expect to observe an enhanced accumulation of phytochemicals in the roots due to increased biosynthesis as well as an increased exudation of phytochemicals from the roots into the nutrient solution. However, no significantly increased accumulation occurred in the illuminated roots for artemisinin in *A. annua* or for secondary compounds in the *H. perforatum* roots or in their exudates. Thus, the enhanced accumulation of secondary compounds observed in the shoot for *A. annua* or *H. perforatum* is unlikely to represent an enhanced biosynthesis of these compounds in the roots. Nevertheless, the effect of root illumination on secondary metabolites in the roots might differ according to plant species. For example, in Arabidopsis, root illumination enhanced the accumulation of secondary metabolites, such as flavonols, in the roots (Silva-Navas et al., 2016; Qu et al., 2017).

The effect of root illumination on root hair formation in our study also differed from the previously reported effect in Arabidopsis roots. In our experiment with *A. annua* and *H. perforatum*, root illumination decreased the frequency of root hair formation, whereas root illumination increased this root hair trait in Arabidopsis. This difference might reflect differences in the cross-talk between ROS, antioxidant biosynthesis, and auxin action in the root epidermis (Garcia-Gonzalez et al., 2021). In *A. annua*, the absence of a clear relationship between root hair formation and the accumulation of secondary compounds in the roots might be at least partly explained by a negative feedback mechanism operating between the accumulation of secondary compounds and root hair formation, indicating no involvement between root hair formation and the modulation of artemisinin content in the roots. Moreover, the strong (3-fold) reduction in root hair density observed in the control plants grown in “water culture” hydroponics was not related to the artemisinin concentration in either the roots or the shoots, based on a comparison with control plants grown under high-pressure aeroponics. A positive correlation between root hair traits and the accumulation of phytochemicals in other plant organs was also absent in *H. perforatum*.

## 5 Outlook

The development of vertical farming opens new opportunities for the control of environmental conditions for the shoot and root systems of cultivated plants. The main aim of cultivation of high-value crops (such as medicinal plants) is to increase the total yield of high-value compounds (Bafort et al., 2022); therefore, optimization of the environmental conditions should include optimization of the elicitors that enhance the accumulation of secondary compounds (phytochemicals) with minimal detrimental effects on plant growth and with a minimal environmental footprint. All these conditions are satisfied by treatments such as root illumination. Light (its intensity and spectrum) is a very promising signal because of its reversibility and its nature-friendly character. Indeed, numerous investigations using *in vitro* culture have shown that light stimulates the biosynthesis of many secondary metabolites (Yoshimatsu et al., 1990; Ramakrishna and Ravishankar, 2011). Further investigation is needed to identify the cross-talk between shoots and roots under the light conditions used in protected agriculture to maximize the accumulation of biologically active compounds in high-value plants without compromising their growth.

## Data availability statement

The original contributions presented in the study are included in the article/**Supplementary Materials**. Further inquiries can be directed to the corresponding author.

## Author contributions

Conceptualization, IP. methodology, IP and MP, validation, MP and IP. formal analysis, MP and IP. investigation, MP, IP, and JZ. resources, IP and MP, data curation, MP. writing—original draft preparation, IP and MP. writing–review and editing, IP, MP and JZ. project administration, MP, funding acquisition, IP. All authors contributed to the article and approved the submitted version.

## Supplementary material

Supplementary Figure 1 The effect of hydroponics **(A, F)**, aeroponics **(B, G)**, and aeroponics with white **(C, H)**, blue **(D, I)**, and red **(E, J)** LED lighting of roots on the size of the aboveground parts of *Artemisia annua* plants **(A-E)** and on root hair formation **(F-J)**. Scale bars represent 10 cm **(A)** and 1 mm **(F)**.
