# Supplementary figures and images for: Light exposure of roots in aeroponics enhances the accumulation of phytochemicals in aboveground parts of the medicinal plants Artemisia annua and Hypericum perforatum
Source: Front Plant Sci. 2023 Jan 19;14:1079656. doi: 10.3389/fpls.2023.1079656 (PMC9893289; doi:10.3389/fpls.2023.1079656)

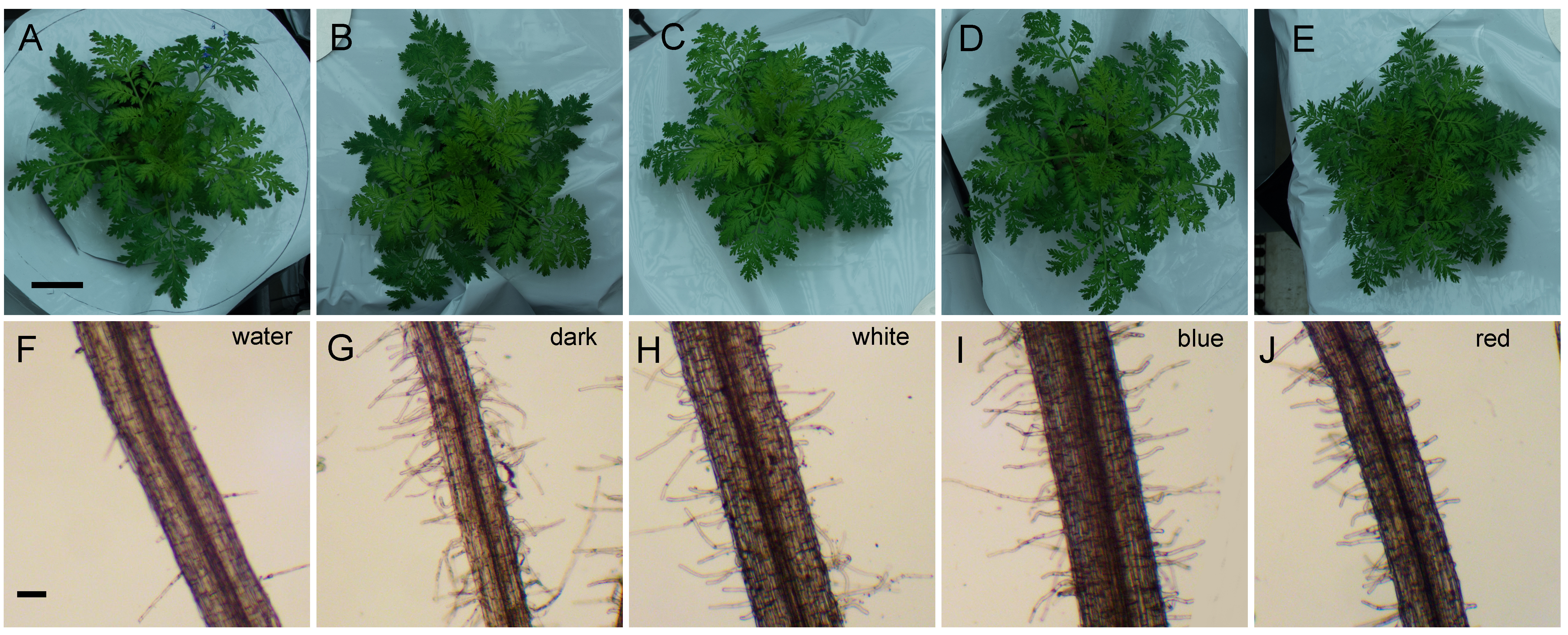

Supplement: Supplementary Figure 1 — The effect of hydroponics (A, F), aeroponics (B, G), and aeroponics with white (C, H), blue (D, I), and red (E, J) LED lighting of roots on the size of the aboveground parts of Artemisia annua plants (A-E) and on root hair formation (F-J). Scale bars represent 10 cm (A) and 1 mm (F). [file Image_1.jpeg]
