# Supplementary material for: Light exposure of roots in aeroponics enhances the accumulation of phytochemicals in aboveground parts of the medicinal plants Artemisia annua and Hypericum perforatum
Source: Front Plant Sci. 2023 Jan 19;14:1079656. doi: 10.3389/fpls.2023.1079656 (PMC9893289; doi:10.3389/fpls.2023.1079656)
Supplement: Supplementary file 2 [file DataSheet_1.pdf]

Suppl. Table 1. MS parameters for MRM-transitions

	MRM transitions	Retention time, min	Declustering potential (DP), V	Entrance potential (EP), V	Cell entrance potential (CEP), V	Collision potential (CE), V	Cell exit potential (CEX), V
Artemisinin	**283→219**	4.5	31	8	14	20	10
	283→151		31	8	14	19	6
	283→209		31	8	14	13	4
	283→265		31	8	14	20	4
	283→247		31	8	14	20	10
	283→229		31	8	14	20	10

Quantifier and qualifier ions are indicated in bold and italics, respectively.
